# Supplementary material for: DeepST: identifying spatial domains in spatial transcriptomics by deep learning
Source: Nucleic Acids Res. 2022 Oct 17;50(22):e131. doi: 10.1093/nar/gkac901 (PMC9825193; doi:10.1093/nar/gkac901)
Supplement: gkac901_Supplemental_Files [file gkac901_supplemental_files.zip › Supplementary Material 1.zip!/SpaGCN_results/Benchmark 4/151673_ARI_0.5274034141902555.pdf]

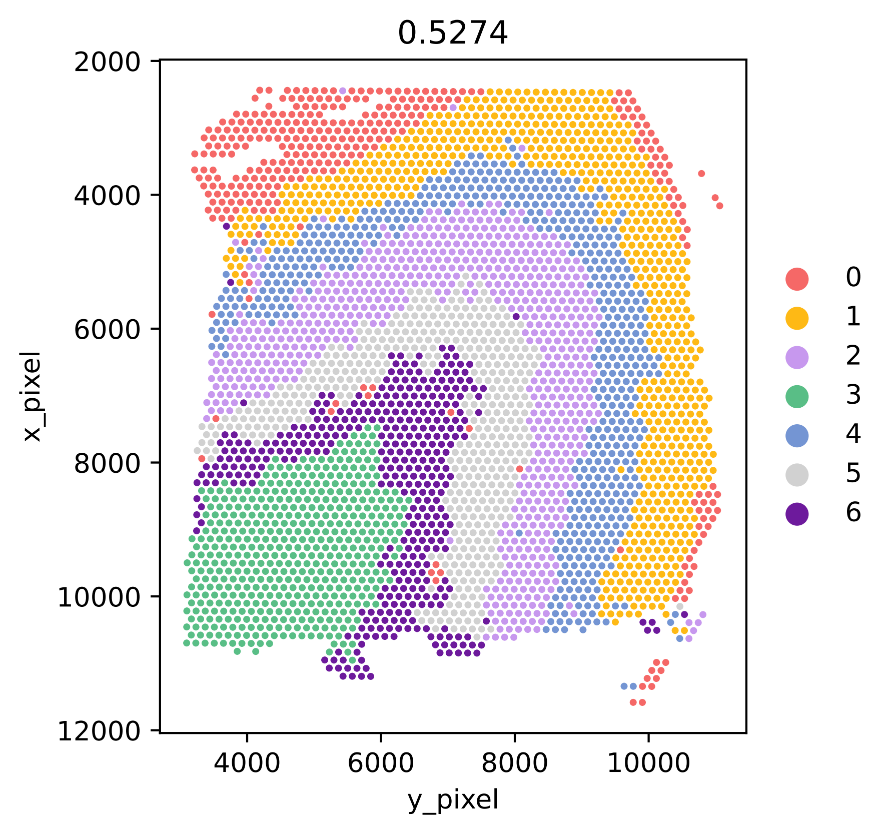
0.5274
2000
4000
6000
8000
10000
12000
x_pixel
4000
6000
8000
10000
y_pixel
0
1
2
3
4
5
6
